# Supplementary material for: Harnessing the open access version of ChatGPT for enhanced clinical opinions
Source: PLOS Digit Health. 2024 Feb 5;3(2):e0000355. doi: 10.1371/journal.pdig.0000355 (PMC10843476; doi:10.1371/journal.pdig.0000355)
Supplement: Attachment — Submitted filename: Response to Reviewers.pdf [file pdig.0000355.s001.pdf]

New York University Grossman Long Island School of Medicine
Mineola, New York 11501
(914) 629-3002
Zachary.Tenner@nyulangone.org

Dear Dr. Silva and Editorial Board,

Thank you very much for taking the time to review our manuscript submission titled *"Harnessing the Open Access Version of ChatGPT for Enhanced Clinical Opinions"*. We greatly appreciate the revisions suggested and have made all considered corrections to the updated version of our manuscript.

Regarding the noted weaknesses of the paper, we've made various grammatical updates to the sentence structure throughout the writing. Additionally, we've added two references to be able to compare our results more effectively in the discussion section. These publications are very helpful to strengthening the conversation around how different versions of generative artificial intelligence can provide differential diagnoses.

We greatly appreciate your positive remarks regarding the introduction and overall presentation of the paper. The proposed revisions were extremely helpful to strengthen our manuscript and we hope raise the manuscript to PLOS Digital Health publication criteria. Please let us know if there are any additional updates that may be helpful for publication.

Please address all correspondence concerning this manuscript to me at Zachary.Tenner@nyulangone.org.

Thank you for your consideration.

Sincerely,

Zachary Tenner
Medical Student
NYU Grossman Long Island School of Medicine
